# The Belgian Association for Psychological Sciences: 75th Anniversary

**DOI:** 10.5334/pb.1140

**Published:** 2022-04-28

**Authors:** Marc Brysbaert

**Affiliations:** 1 Department of Experimental Psychology, Ghent University, B-3000 Gent, Belgium

**Keywords:** learned society, Belgium, history, Belgian Association for Psychological Sciences

## Abstract

The Belgian Association for Psychological Sciences (BAPS) is a learned society founded in 1947. Its mission is to unite people in Belgium interested in the development and application of psychological sciences. It does so through the publication of Psychologica Belgica, the organisation of an annual scientific meeting, the award of prizes, initiatives to improve the communication among members, and representing researchers and psychologists nationally and internationally. The present paper describes the third 25-year period of BAPS. It reviews the main initiatives and activities of the society from 1997 to 2022.

The Belgian Association for Psychological Sciences (or the Belgische Vereniging voor Psychologie/Société Belge de Psychologie, as it was called then) was established in 1947. The minutes of the initial meetings can be found in the first volume of Psychologica Belgica, published in 1954 (as illustrated in ***Figures 1, 2, 3***; see also supplementary materials). A short review of the first 50 years of the society is available in Brysbaert and Schets (1997). The present article covers the third period of 25 years, from 1997 to 2022.

**Figure 1:**
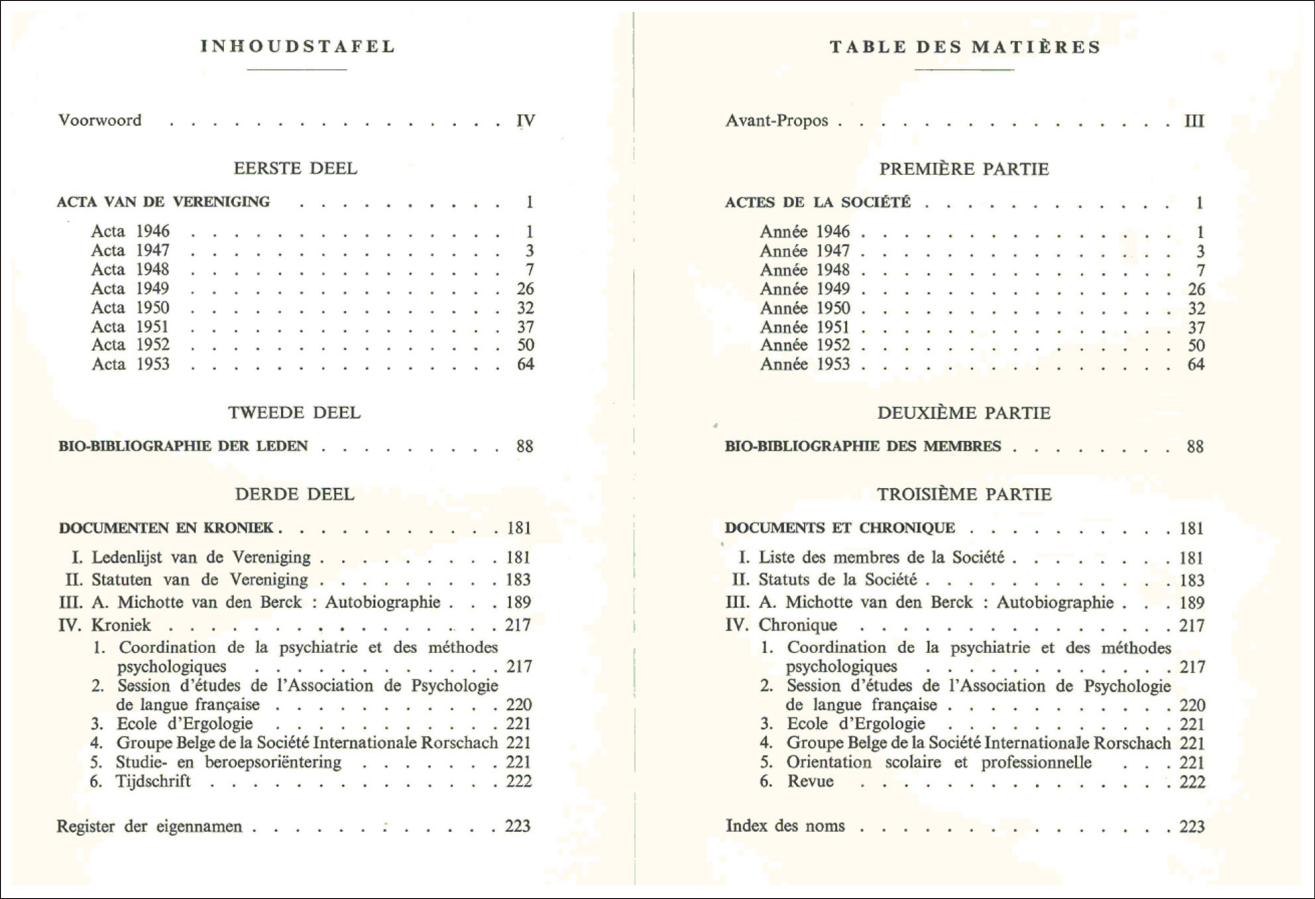
Table of contents of the first volume of Psychologica Belgica. This volume was published in 1954 and included, among other items, the summaries of the meetings in the first years, the society’s member list, and the statutes of the society. Notice that everything was written in Dutch and in French, the two languages in which psychology was taught.

**Figure 2:**
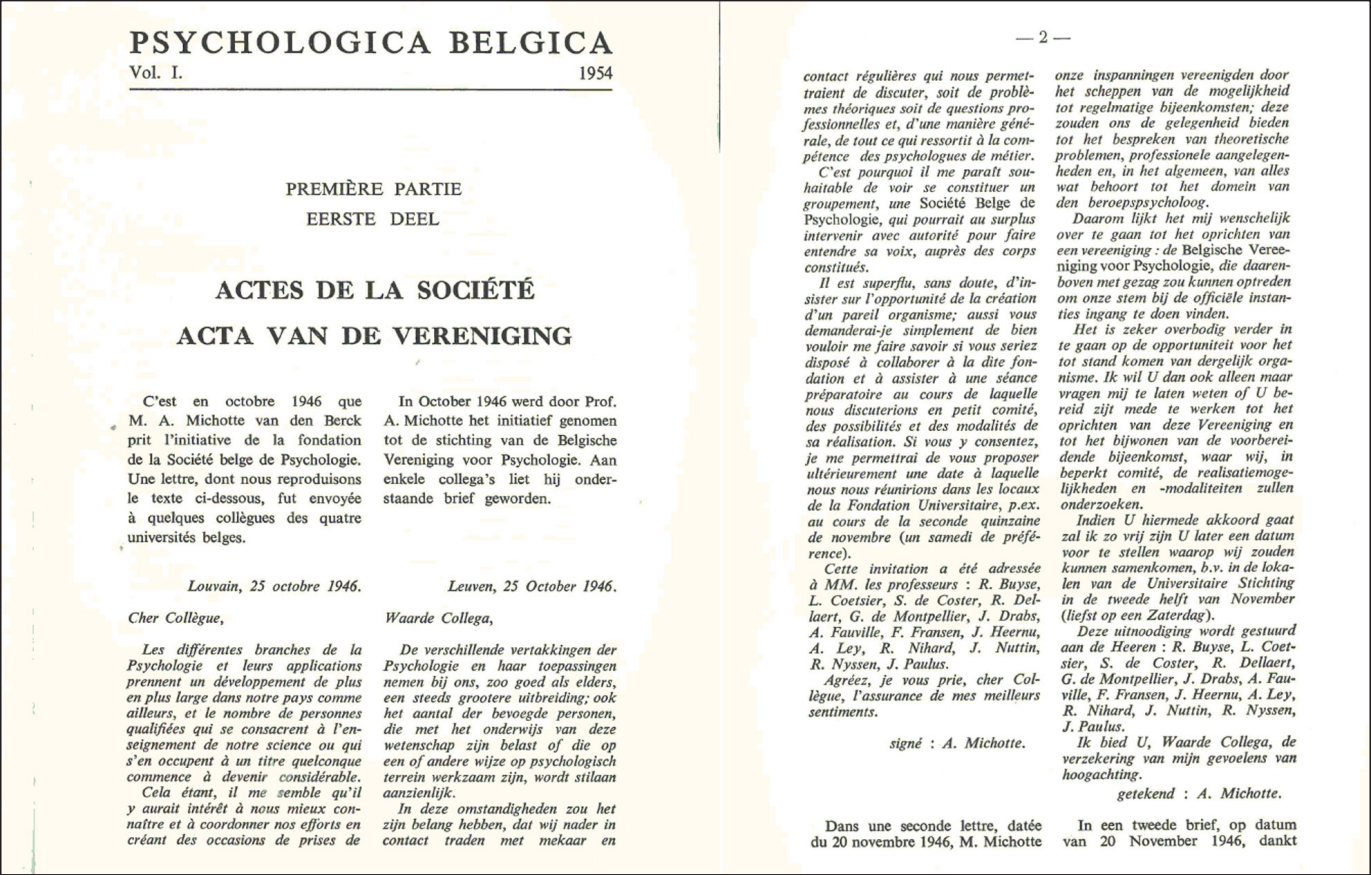
A copy of the letter of invitation sent by Michotte to colleagues to start a learned society for researchers in psychology (as printed in the first volume of Psychologica Belgica).

**Figure 3:**
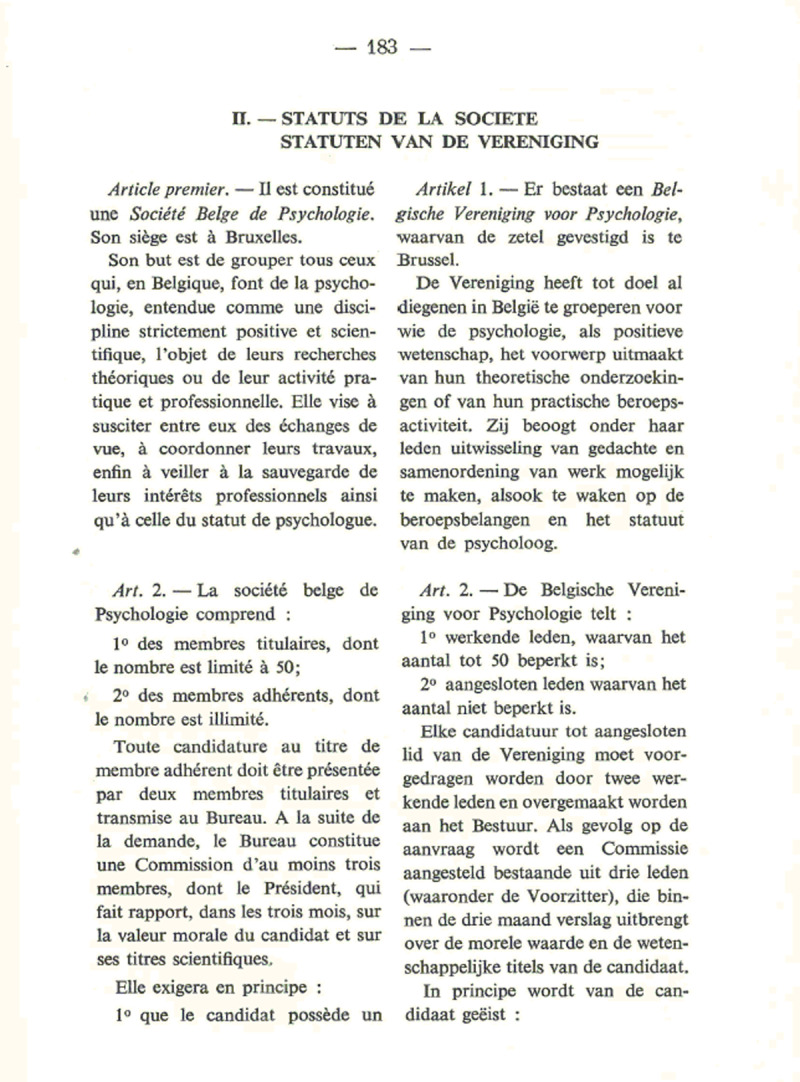
Extract of the society’s bylaws (as published in the first volume of Psychologica Belgica).

## The legal status of the society

The mission of the Belgian Association for Psychological Sciences (BAPS) is to unite all those in Belgium interested in the development and application of psychological sciences. In the first 50 years, members were predominantly academics teaching at universities. This changed towards the end of the 20^th^ century, when 29 European countries expressed a desire to harmonize the European higher education area by the creation of 3-year bachelor degrees and 1- to 2-year master’s degrees (an initiative known as the Bologna Declaration, 1999). As a result, bachelor degrees of applied psychology, taught at Hogescholen or Instituts Supérieurs, became integrated in the university system, with a larger focus on research. At that time, BAPS welcomed lecturers teaching in these degrees as well.

The legal status of BAPS was changed in 2005. Before, the society was largely informal, without a clear legal structure and responsibilities. Under initiative of André Vandierendonck, the society took the form of a non-profit organisation (vereniging zonder winstoogmerk/association sans but lucratif). This required explicit definition of the society’s mission, which was an opportunity to update the statutes. One of the changes was that the society would use English as the official language of communication, instead of Dutch and French. As a result, the name changed to BAPS (with some discussion as to whether the scientific character of the society should be defined as a unitary approach (science) or a pluralistic enterprise (sciences), with the latter being adopted in the end).^1^

The following were the main features of BAPS according to its 2005 constitution charter:

– The Belgian Association of Psychological Sciences is a non-profit organisation which has the mission to promote and to support scientific psychology in Belgium.
– The society deploys the following activities to achieve its goals: (1) It publishes the internationally distributed scientific journal, Psychologica Belgica, of which the society possesses the title, (2) it organises regular scientific meetings to encourage exchange and discussion of new findings and ideas and to help young researchers in psychology to make contacts with other researchers in the field, (3) it organises annually the BAPS best thesis award for the best master’s thesis, which contributes to psychological science and is realised at one of the Belgian universities with a master curriculum in psychology, and (4) the society encourages publication of research findings and creates the possibilities to improve communication among its members and between members and other interested parties.
– The society distinguishes three types of members: full members, associate members and affiliate members. Only full members have the right to vote at the General Assembly. The associate and affiliate members are joined members. Full membership is open to persons with an academic diploma of doctor in psychology or psychological sciences. Persons with an academic diploma of doctor in another scientific discipline can apply for full membership on condition that their scientific work is connected to psychology. Persons with a master’s diploma in psychology can apply for associate membership. After a term of minimally 6 years of associate membership, associate members can become full members. Persons with a bachelor degree can apply for affiliate membership.
– The society is administered by an Executive Committee. This committee consists of minimally five and maximally nine members. These are: the president, the past president, the secretary, the treasurer, the journal editor, and two to four other persons who take responsibility for the management of particular initiatives of the society. No more than two of these members can belong to the same institution or university; no more than two thirds of the members can belong to same linguistic community. In order to take valid decisions, at least three members of the Administrative Council must be present.
– All members of the Executive Committee are appointed by the General Assembly for a period of three years. This period normally starts the first of July following the General Assembly meeting in May and ends 30 June three years later.
– At the end of a three-year term of service, the president automatically becomes the next past-president. After ending the three-year term as past-president this person is not immediately eligible again for membership of the executive committee.

The constitution was updated in 2020. The main changes were:

– Change of membership categories into full member, PhD student, and master student.
– Increase of the Executive Committee to minimally nine and maximally fifteen members, in order to have delegates from the seven Belgian universities awarding a master degree in psychology and a delegate from the non-university colleges offering degrees in applied psychology.
– The inclusion of new awards granted by the Society, such as the poster prizes, best PhD thesis award, and early career award (discussed below).
– Update of the statutes to make them conform to changes in Belgian law.

The revision further aimed to make the society more inclusive. This involved the inclusion of all fields of psychology within the Executive Committee, and a larger diversity in the selection of invited symposia and keynote lectures at the annual meetings. There was an effort to improve the gender balance with for the first time a majority of women in the Executive Committee of 2021 (6 members out of 11).

## Psychologica Belgica

Publication of Psychologica Belgica is listed as the first commitment of the Society. The first volume was published in 1954 (making it one of the oldest journals in psychology). For a long time, the journal was published by Leuven University Press, with administrative help from the Faculty of Psychology and Educational Sciences at the University of Leuven. Publication costs increased year on year and when in 2005 the Faculty in Leuven indicated it was no longer able to provide the administrative support, it was decided to search for a publisher with better value for money (Verfaillie, 2006). This was found in the Ghent publisher, Academia Press, that printed and distributed the journal from 2006 till 2013. In addition, the articles were made available in portable document format (pdf) via Ingenta Connect, so that they could be downloaded worldwide.

Changes in the distribution of the journal and in the ways authors, editors and reviewers interacted (with increasing reliance on the internet) meant that the journal started to fall outside the core business of the publisher in the early 2010s. Around that time the society was contacted by a new British publisher of open access journals, Ubiquity Press, which was a perfect match for the society’s ambitions to make research findings easily available (Van Hiel, 2014; Majerus, 2018).

Psychologica Belgica was one of the first traditional journals to go open access, with an average article processing cost lower than the production costs. For Ubiquity Press, Psychologica Belgica was their first journal with a listing in the Web of Science. Distribution of the journal became fully online, with articles that could be downloaded for free (via *https://www.psychologicabelgica.com/*). BAPS made an extra investment to scan all old articles going back to 1975 (Volume 15) and make them freely available via the website. In 2019 the journal introduced a new section called *PhD Critical Review* papers, to provide a platform for young PhDs to publish the theoretical parts of their PhD thesis, in the form of a theoretical review paper.

Psychologica Belgica publishes 10–30 articles per year (Majerus, 2021). The titles of the five most cited articles give an impression of the type of articles published in the journal. They are a mix of general research topics and applied research within the Belgian context (e.g., the development of research tools in Dutch and French).

The titles of the five most cited articles are:

– Combining speed and accuracy in cognitive psychology: Is the inverse efficiency score (IES) a better dependent variable than the mean reaction time (RT) and the percentage of errors (PE)? (Bruyer & Brysbaert, 2011)
– Does working memory training generalize? (Shipstead et al., 2010)
– Measuring empathic tendencies: Reliability and validity of the Dutch version of the Interpersonal Reactivity Index (De Corte et al., 2007)
– Affective and subjective familiarity ratings of 740 Dutch words (Hermans & De Houwer, 1994)
– Exploring the causes of comparative optimism (Shepperd et al., 2002)

These were the editors of the journal since 1997 (see Brysbaert & Schets, 1997, for the earlier editors):

– André Vandierendonck (1994–1999)
– Axel Cleeremans (2000–2005)
– Karl Verfaillie (2006–2010)
– Etienne Quertemont (2011–2013)
– Alan Van Hiel (2014–2017)
– Steve Majerus (2018–)

## Annual meeting

The second objective of BAPS is to organise an annual meeting, where researchers can present their latest findings and interact with colleagues. The meetings are organised at universities to keep costs low. In line with the increased number of psychology researchers, attendance has grown over the years and the traditional one-day meeting became quite packed, with up to 8 parallel sessions and large poster sessions (strategically placed in the lunch and reception room to have maximal attendance). The number of plenary sessions with invited speakers also needed to be limited to 2 at most. At the 2019 annual meeting in Liège the traditional one-day meeting was extended by a dinner and an additional half day during which local organizers facilitated the meeting of sub-groups of researchers. This is an initiative the Society plans to repeat, which may become the stepping stone to a two-day meeting.

The annual meeting typically takes place at the end of May. There is a rotation between universities, with occasional exceptions. One exception was the meeting of 2016 at the Thomas More University College in Antwerp. Another was held at the Palace of the Royal Academies of Belgium in 2017. The Louvain meeting in 2020 had to be cancelled and replaced by a half-day online meeting because of the Covid19 lockdown (it was one of the first psychology meetings offering an online format). The Louvain organisers kindly volunteered to organise the 2021 meeting too, assuming this would be an in-person meeting, and saw themselves forced to organise a full online meeting with an invited speaker and symposia because of the prolonged lockdown. An innovation at that meeting was the inclusion of an interactive poster session with software that allowed participants to walk through the different poster rooms and ask questions directly to the poster presenters.

In 2002 BAPS organised a joint three-day meeting with the British Experimental Psychology Society (in Leuven), which had a very large attendance. On this occasion, an Honorary Fellowship was awarded to Glynn Humphreys for his many initiatives to foster collaboration between researchers in Belgium and the UK. In 2007 the society was invited back to a joint EPS meeting in Cardiff. Another three-day joint meeting was held in Liège (2012) with the Sociedad Española de Psicología Experimental (SEPEX).

Since 2003, BAPS sponsors three prizes for best poster presented at the annual meeting. The purpose of the award is to foster creative poster design, to reward effective poster presentations, and to recognize the importance of poster presentations at the annual meeting. Posters are judged by a panel of three members appointed by the Executive Committee.

Increasingly, satellite meetings became organised around the annual meeting. These are primarily aimed at young researchers (mostly organised by young researchers as well) dealing with practical and ethical matters in research. For instance, the Junior Board organised an online satellite meeting at the 2021 Louvain meeting.

In 2022 BAPS intends to organize a special two-day meeting (in Leuven) to celebrate the 75^th^ anniversary.

## Prizes and awards

To encourage psychological research and reward promising students, BAPS started with a master’s thesis award in 1995. The winner and runner-ups are announced at the annual meeting.

In the 2010–2020s three new awards were initiated: Best Bachelor’s Thesis Award (2016–2020), Best PhD thesis award (2018), and Early Career Award (2020). The addition of these prizes was one of the reasons for the revision of the statutes in 2020 (so that the prizes were included).

The best master’s thesis and PhD thesis awards follow a two-step evaluation procedure. First, candidates are evaluated on the basis of a summary of the thesis, an indication of the impact of the work, and (for the PhD thesis) the applicant’s CV. This step is done by members of the BAPS executive committee. It results in a shortlist, which is evaluated by an independent panel. For the master’s thesis award this is organised by the National Committee of Psychological Sciences at the Royal Academy of Belgium, whose members kindly agreed to be jury members. For the PhD thesis award, the jury consists of BAPS past-presidents and experts well-acquainted with the research domains of the shortlisted candidates. The first winners of the PhD prize were: Kobe Desender (2018), Jasper Van Assche (2019), and Dries Bostyn (2021).

The early career award was launched to recognize the scientific excellence of researchers in psychological sciences up to 6 years after their PhD and working at a Belgian research institution. The award is conferred every two years. The applications are assessed by a jury of international senior researchers familiar with the topics of the applicants. The jury members are suggested to BAPS by the national associations of psychology that are active in the Board of Scientific Affairs of EFPA (European Federation of Psychologists’ Associations). In 2020, the jury was composed of 5 members with expertise in neurosciences and in clinical, cognitive, health, and social psychology coming from Germany, Norway, Spain, Sweden, and the UK. The first laureate was Pieter Van Dessel (in 2020).

## Further initiatives

The last 25 years saw other new initiatives taken. Arguably the most salient was the establishment of a Junior Board in 2019. The mission of the Junior Board is to bring together junior scholars (master, PhD, post-doc) working in Belgium. It aims to represent and serve junior scholars. The Junior Board adheres to the same principles as the BAPS Executive Committee with which it interacts closely. It consists of elected members from higher education institutes where psychology research takes place.

The two main goals of the Junior Board are 1) to foster scientific collaboration between junior and senior researchers, mainly at a national level, and 2) to address the specific difficulties junior researchers may face during their early academic careers, in particular topics concerning the well-being of junior researchers and their integration within and outside of academia. One delegate of the junior board participates in the meetings of the Executive Committee. The junior board is also in close contact with the Belgian Federation for Psychology Students (BFSP).

The Junior Board aims to promote Open Science within the field of Psychology, and to raise awareness about researchers’ ecological footprint, especially when travelling abroad. The Junior Board further reflects on how new (social) communication tools (e.g., Facebook, Twitter, blogs) can help to disseminate scientific research to the broader scientific community and the general public. The organisation of the first Junior Day in 2021 with recorded talks on well-being in academia, Open Science and scientific communication was one of the first main activities of the Junior Board. Another initiative was the organisation of webinars about well-being and environmental psychology.

**Figure 4:**
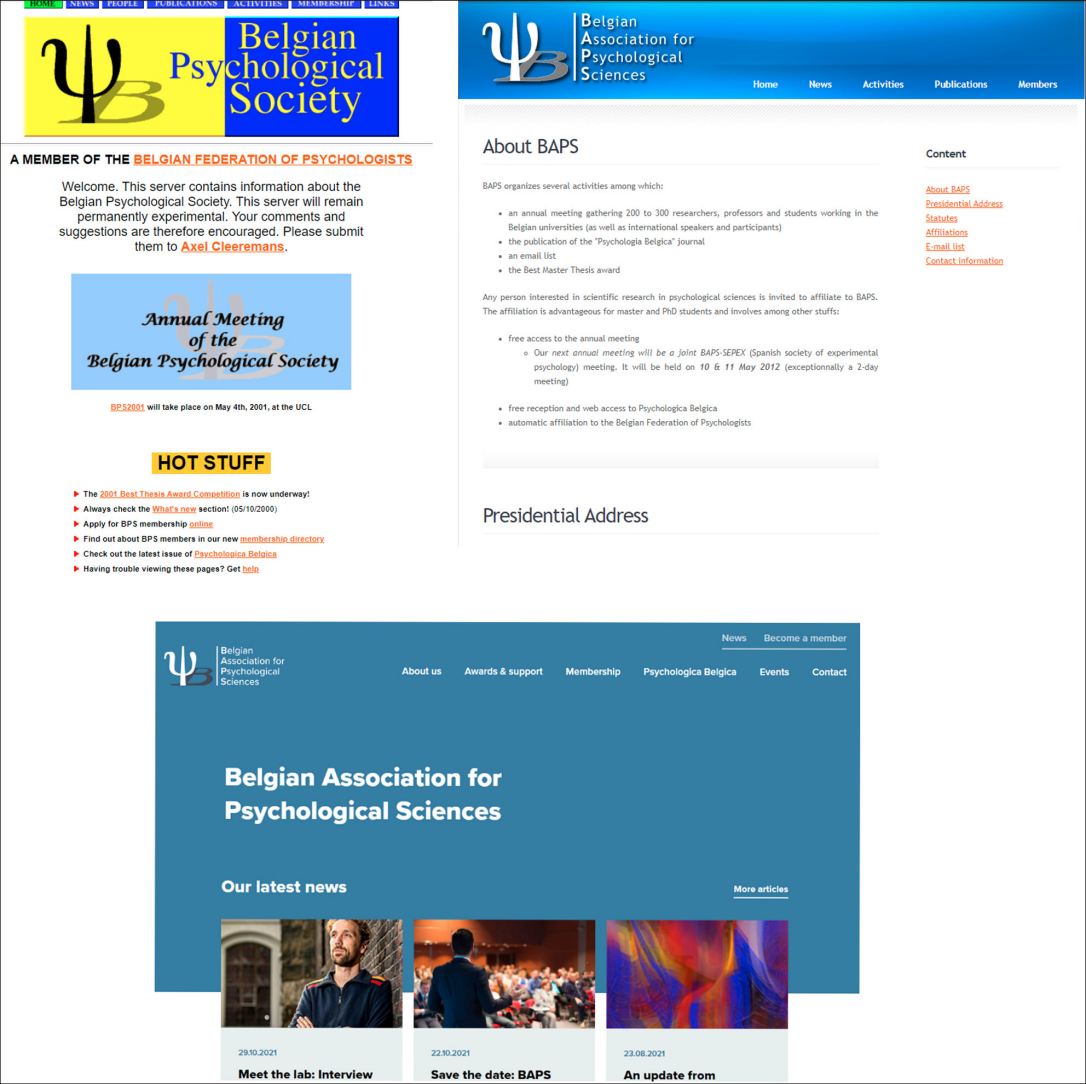
Screenshots of the BAPS website in 2001, 2011, and 2021 (from top to down).

Another new initiative was the start of a mailing list by Frank Van Overwalle in 2004. Everyone interested can easily subscribe to the mailing list and unsubscribe. Approved members can send messages (in order to prevent spam messages). In practice the mailing list is predominantly used to announce job openings and for communication with the members. In addition to the mailing list, the society started a website (*https://www.baps.be/*), where recent information about the society and upcoming events can be found. The website got several overhauls (***Figure 4***), the most recent in October 2021 as part of the preparations for the 2022 anniversary celebrations.

As part of the 2020 statutes revision, the decision was taken to add other types of communication. In particular, the initiative was taken to launch a monthly electronic newsletter, which includes job offers, relevant trainings and meetings, calls for awards, recently accepted papers in Psychologica Belgica, and interviews with heads of Belgian research groups and award winners. It is possible that in the future the newsletter will replace the mailing list. To streamline communication, a member was added to the Executive Committee, specifically responsible for communication (including social media).

To better connect Belgian researchers, BAPS started to sponsor workshops and meetings, which contribute to the development of scientific psychology and its applications in Belgium. The society also took the initiative to facilitate research internships between Belgian universities. Supervisors can post research internship positions and attract students from other Belgian universities in order to facilitate the mobility of the upcoming psychology researchers.

In response to the Covid19 epidemic, BAPS became more active in promoting the societal impact of psychological sciences. One member of the executive committee was dedicated to this action. The society helped establish a group of academics and representatives of professional psychology to create a “psychology and corona” expert group in the summer of 2020 with the aims:

- To advance a conceptual framework on corona-related behaviour as a basis for strategic advice
- To collect and disseminate information on ongoing research and existing databases
- To write and distribute regular short reports with practical and concrete do’s/don’ts
- To develop visibility and building up a direct relationship with media/journalists and committees of other experts and policy makers
- To become the contact point for questions by media and policy makers on corona-related behaviour

The group included a BAPS delegate and was perceived as successful in achieving its goals. Following this initiative, BAPS launched a list of experts to provide easy access to specific expertise in psychological knowledge in Belgium, and to facilitate the work of political authorities, media, professional psychologists and other associations.

## Belgian Federation of Psychologists

BAPS is a founding member of the Belgian Federation of Psychologists (BFP), an organisation to defend and promote the rights of psychologists practising in Belgium. Because of changed legislation and new structures, the Federation needed major adjustments in 2020, for which BAPS took a leading role. BAPS delegates were instrumental in coordinating the Quo Vadis group to accompany the BFP from a federation of individual members to a federation of member organisations. The new BFP has three central goals: to improve psychologists’ rights at the federal level, to coordinate the representation of Belgian delegates at the European Federation of Psychologists’ Associations (EFPA), and to coordinate the communication of Belgian psychologists. BAPS has also delegates at the Commission of Psychologists (Compsy, dealing with legal aspects of psychologists). They represent the sector of academic research.

## International links

BAPS is connected to several international associations for the promotion of psychological sciences. In 2021 a BAPS delegate became member of the Board of Scientific Affairs of EFPA (European Federation of Psychology Associations). BAPS is also active in organising symposia at ICPS conferences, and has a delegate at the International Union of Psychological Science (IUPSYS).

## Officers of BAPS

Brysbaert and Schets (1997) listed the 19 presidents up to 1999. Since, the following people have taken over:

– Frank Van Overwalle (VUB Brussel 1999–2002)
– André Vandierendonck (UGhent, 2002–2005)
– Axel Cleeremans (ULB Bruxelles, 2005–2008)
– Marc Brysbaert (UGhent, 2008–2011)
– Steve Majerus (ULg Liège, 2011–2014)
– Johan Wagemans (KU Leuven, 2014–2017)
– Olivier Luminet (UC Louvain, 2017–2020)
– Jan De Houwer (UGhent, 2020–2023)

There has been a strict alternation between presidents from universities in the Dutch-speaking and French-speaking parts of the country. Additional attempts were made to find diversity in universities and gender, but certainly as far as gender is concerned these attempts were not successful, a challenge for future committees.

The honorary secretaries since 1997 were:

– Vincent Yzerbyt (UC Louvain) & Frank Van Overwalle (VUB Brussel) (1996–1999)
– Jean-Pierre Thibaut (ULg Liège) & Vera Hoorens (KU Leuven) (1999–2002)
– Olivier Klein (ULB Bruxelles) & Karl Verfaillie (KU Leuven) (2002–2004)
– Olivier Klein (ULB Bruxelles, 2005–2008)
– Emmanuelle Zech (UC Louvain, 2008–2011)
– Alain Van Hiel (UGhent, 2011–2014)
– Dana Samson (UC Louvain, 2014–2016)
– Kasia Uzieblo (Thomas More, 2016–2017)
– Alan Van Hiel (UGhent, 2017–2020)
– Gilles Vannuscorps (UC Louvain, 2020–)

The treasurers since 1997 were:

– Robert Schets (1987–2002)
– Tom Beckers (KU Leuven, 2002–2008)
– Bert Reynvoet (KU Leuven, 2008–2014)
– Eva Van den Bussche (VUB Brussel, KU Leuven, 2014–2020)
– Dinska Van Gucht (Thomas More, KU Leuven, 2020–)

## To the 100th anniversary

Having passed the age of 75, BAPS is now on its way to its 100th anniversary. A comparison of the current overview with that of Brysbaert and Schets (1997) simultaneously illustrates the continuity of the society and its agility to seize new opportunities. BAPS has provided a warm home for psychology researchers in Belgian universities and plans to continue that role. Psychology has experienced significant growth since the society’s early days and is likely to do so in the coming decades, providing fertile ground for the society’s further progress.

## Additional Files

The parts of the first volume of Psychologica Belgica related to the establishment of BAPS have been scanned in and are made available as supplementary materials.

10.5334/pb.1140.s1 Documents related to the BAPS foundation Part 1.

10.5334/pb.1140.s2 Documents related to the BAPS foundation Part 2.

## Footnotes

Some remnants of the discussion can be found in the foundation application, where the plural is used in the French and Dutch names, but the singular in the English name.
