# Supplementary material for: The Belgian Association for Psychological Sciences: 75th Anniversary
Source: Psychol Belg. 2022 Apr 28;62(1):184–92. doi: 10.5334/pb.1140 (PMC9053530; doi:10.5334/pb.1140)
Supplement: Documents related to the BAPS foundation Part 1. [file pb-62-1-1140-s1.pdf]

2A / 47

PSYCHOLOGICA BELGICA

ANNALES DE LA SOCIÉTÉ BELGE DE PSYCHOLOGIE

ANNALEN VAN DE BELGISCHE VERENIGING VOOR PSYCHOLOGIE

Vol. I
1946-1953

RIJKSUNIVERSITEIT GENT
LABORATORIUM
VOOR
TOEGEPASTE PSYCHOLOGIE
COUPURE, 86 - GENT

ÉDITIONS E. NAUWELAERTS
2, Place Cardinal Mercier
LOUVAIN

UITGAVEN E. NAUWELAERTS
Kardinaal Mercierplein 2
LEUVEN

1954

UNIVER
SITEITS
BIBLIO

INHOUDSTAFEL

Voorwoord IV

EERSTE DEEL

ACTA VAN DE VERENIGING 1

Acta 1946 1
Acta 1947 3
Acta 1948 7
Acta 1949 26
Acta 1950 32
Acta 1951 37
Acta 1952 50
Acta 1953 64

TWEEDE DEEL

BIO-BIBLIOGRAPHIE DER LEDEN 88

DERDE DEEL

DOCUMENTEN EN KRONIEK 181

I. Ledenlijst van de Vereniging 181
II. Statuten van de Vereniging 183
III. A. Michotte van den Berck : Autobiographie . . . 189
IV. Kroniek 217
1. Coordination de la psychiatrie et des méthodes psychologiques 217
2. Session d'études de l'Association de Psychologie de langue française 220
3. Ecole d'Ergologie 221
4. Groupe Belge de la Société Internationale Rorschach 221
5. Studie- en beroepsoriëntering 221
6. Tijdschrift 222

Register der eigennamen 223

TABLE DES MATIÈRES

Avant-Propos III

PREMIÈRE PARTIE

ACTES DE LA SOCIÉTÉ 1

Année 1946 1
Année 1947 3
Année 1948 7
Année 1949 26
Année 1950 32
Année 1951 37
Année 1952 50
Année 1953 64

DEUXIÈME PARTIE

BIO-BIBLIOGRAPHIE DES MEMBRES 88

TROISIÈME PARTIE

DOCUMENTS ET CHRONIQUE 181

I. Liste des membres de la Société 181
II. Statuts de la Société 183
III. A. Michotte van den Berck : Autobiographie . . . 189
IV. Chronique 217
1. Coordination de la psychiatrie et des méthodes psychologiques 217
2. Session d'études de l'Association de Psychologie de langue française 220
3. Ecole d'Ergologie 221
4. Groupe Belge de la Société Internationale Rorschach 221
5. Orientation scolaire et professionnelle . . . 221
6. Revue 222

Index des noms 223

VOORWOORD

De Belgische Vereniging voor Psychologie publiceert in dit Jaarboek de *Acta* van de *Vereniging* sedert haar ontstaan in 1946 tot December 1953. In een tweede hoofdstuk van het boek vindt men het *Curriculum vitae* en de Bibliographie van de leden van de Vereniging.

Aangezien de Vereniging de meeste universitaire psychologen in België onder haar leden telt, geeft deze *Bibliographie* een vrij goed overzicht van de psychologische publicaties in ons land gedurende de laatste jaren.

Daarenboven worden in een derde hoofstuk van deze aflevering documenten en kronieken gepubliceerd in betrekking met het psychologisch leven in België. In dit eerste Jaarboek hebben wij het voorrecht voor het eerst de originele franse tekst te kunnen publiceren van een hoofdstuk uit de *History of Psychology in Autobiography*. Het betreft namelijk het leven en werk van Professor A. Michotte van den Berck, Stichter en Ere-Voorzitter van onze Vereniging.

Het ligt in de bedoeling van het Bestuur van de Vereniging van tijd tot tijd een aflevering zoals dit Jaarboek te laten verschijnen, waarin een beeld zal worden opgehangen van de wetenschappelijke activiteit op psychologisch gebied in ons land. Daarom hebben wij gemeend aan deze publicatie de titel te mogen geven van : *Psychologica Belgica.*

Wij danken ten zeerste alle leden van de Vereniging die zo bereidwillig waren ons de nodige inlichtingen voor de samenstelling van dit eerste Jaarboek mede te delen.

J. NUTTIN,
Secretaris van de Belgische Vereniging voor Psychologie.

AVANT-PROPOS

La Société belge de Psychologie publie, dans une première partie de ce volume, les actes de la Société depuis sa fondation, en 1946, jusqu'en décembre 1953. Dans une seconde partie, on trouve la bio-bibliographie des membres.

Etant donné que la Société groupe la plupart des psychologues universitaires en Belgique, la liste bibliographique de ses membres donnera un aperçu assez complet des publications scientifiques belges dans le domaine de la psychologie.

Dans une troisième partie du volume, quelques documents et chroniques concernent la Société et la psychologie en Belgique. Nous avons le plaisir de faire paraître ici, pour la première fois, le texte français original d'un chapitre de la *History of Psychology in Autobiography*. Il s'agit de la vie et de l'œuvre du Professeur A. Michotte van den Berck, Fondateur et Président d'Honneur de notre Société.

Le Bureau de la Société se propose de publier périodiquement un volume comme celui-ci où il sera donné un aperçu de l'activité psychologique en Belgique en rapport avec l'activité de la Société. C'est le motif pour lequel nous avons pensé pouvoir donner à ces volumes le titre *Psychologica Belgica.*

Nous remercions très vivement tous les membres de la Société qui ont bien voulu nous fournir les renseignements nécessaires pour ce premier volume.

J. NUTTIN,
Secrétaire de la Société belge de Psychologie.

PSYCHOLOGICA BELGICA

Vol. I. 1954

PREMIÈRE PARTIE

EERSTE DEEL

ACTES DE LA SOCIÉTÉ

ACTA VAN DE VERENIGING

C'est en octobre 1946 que M. A. Michotte van den Berck prit l'initiative de la fondation de la Société belge de Psychologie. Une lettre, dont nous reproduisons le texte ci-dessous, fut envoyée à quelques collègues des quatre universités belges.

In October 1946 werd door Prof. A. Michotte het initiatief genomen tot de stichting van de Belgische Vereniging voor Psychologie. Aan enkele collega's liet hij onderstaande brief geworden.

Louvain, 25 octobre 1946.

Cher Collègue,

Les différentes branches de la Psychologie et leurs applications prennent un développement de plus en plus large dans notre pays comme ailleurs, et le nombre de personnes qualifiées qui se consacrent à l'enseignement de notre science ou qui s'en occupent à un titre quelconque commence à devenir considérable.

Cela étant, il me semble qu'il y aurait intérêt à nous mieux connaître et à coordonner nos efforts en créant des occasions de prises de

Leuven, 25 October 1946.

Waarde Collega,

De verschillende vertakkingen der Psychologie en haar toepassingen nemen bij ons, zoo goed als elders, een steeds grootere uitbreiding; ook het aantal der bevoegde personen, die met het onderwijs van deze wetenschap zijn belast of die op een of andere wijze op psychologisch terrein werkzaam zijn, wordt stilaan aanzienlijk.

In deze omstandigheden zou het zijn belang hebben, dat wij nader in contact traden met mekaar en

contact régulières qui nous permettraient de discuter, soit de problèmes théoriques soit de questions professionnelles et, d'une manière générale, de tout ce qui ressortit à la compétence des psychologues de métier.

C'est pourquoi il me paraît souhaitable de voir se constituer un groupement, une Société Belge de Psychologie, *qui pourrait au surplus intervenir avec autorité pour faire entendre sa voix, auprès des corps constitués.*

Il est superflu, sans doute, d'insister sur l'opportunité de la création d'un pareil organisme; aussi vous demanderai-je simplement de bien vouloir me faire savoir si vous seriez disposé à collaborer à la dite fondation et à assister à une séance préparatoire au cours de laquelle nous discuterions en petit comité, des possibilités et des modalités de sa réalisation. Si vous y consentez, je me permettrai de vous proposer ultérieurement une date à laquelle nous nous réunirions dans les locaux de la Fondation Universitaire, p.ex. au cours de la seconde quinzaine de novembre (un samedi de préférence).

Cette invitation a été adressée à MM. les professeurs : R. Buyse, L. Coetsier, S. de Coster, R. Dellaert, G. de Montpellier, J. Drabs, A. Fauville, F. Fransen, J. Heernu, A. Ley, R. Nihard, J. Nuttin, R. Nyssen, J. Paulus.

Agréez, je vous prie, cher Collègue, l'assurance de mes meilleurs sentiments.

signé : A. Michotte.

onze inspanningen vereenigden door het scheppen van de mogelijkheid tot regelmatige bijeenkomsten; deze zouden ons de gelegenheid bieden tot het bespreken van theoretische problemen, professionele aangelegenheden en, in het algemeen, van alles wat behoort tot het domein van den beroepspsycholoog.

Daarom lijkt het mij wenschelijk over te gaan tot het oprichten van een vereeniging : de Belgische Vereeniging voor Psychologie, *die daarenboven met gezag zou kunnen optreden om onze stem bij de officiële instanties ingang te doen vinden.*

Het is zeker overbodig verder in te gaan op de opportuniteit voor het tot stand komen van dergelijk organisme. Ik wil U dan ook alleen maar vragen mij te laten weten of U bereid zijt mede te werken tot het oprichten van deze Vereeniging en tot het bijwonen van de voorbereidende bijeenkomst, waar wij, in beperkt comité, de realisatiemogelijkheden en -modaliteiten zullen onderzoeken.

Indien U hiermede akkoord gaat zal ik zo vrij zijn U later een datum voor te stellen waarop wij zouden kunnen samenkomen, b.v. in de lokalen van de Universitaire Stichting in de tweede helft van November (liefst op een Zaterdag).

Deze uitnoodiging wordt gestuurd aan de Heeren : R. Buyse, L. Coetsier, S. de Coster, R. Dellaert, G. de Montpellier, J. Drabs, A. Fauville, F. Fransen, J. Heernu, A. Ley, R. Nihard, J. Nuttin, R. Nyssen, J. Paulus.

Ik bied U, Waarde Collega, de verzekering van mijn gevoelens van hoogachting.

getekend : A. Michotte.

Dans une seconde lettre, datée du 20 novembre 1946, M. Michotte remercie ses Collègues de leur accord unanime et convoque une première réunion dans les locaux de la Fondation Universitaire, 11 rue d'Egmont à Bruxelles, en vue d'une première prise de contact.

In een tweede brief, op datum van 20 November 1946, dankt Prof. Michotte zijn collega's voor hun eenstemmige toetreding en belegt een eerste samenkomst in de lokalen van de Universitaire Stichting, Egmontstraat 11, te Brussel, met het oog op een eerste contactname.

Séance du **Vergadering van**

30-XI-1946

Présents : MM. Buyse, Coetsier, De Coster, Dellaert, Drabs, Fauville, Fransen, Heernu, Ley, Michotte, de Montpellier, Nihard, Nuttin, Nyssen, Paulus.

M. Michotte, président, ouvre la séance à 10 h. Il expose le but de la réunion : la constitution d'une Société belge de Psychologie groupant les spécialistes des quatre universités et accessible à ceux qui s'adonnent à cette discipline d'une manière positive. Une association de ce genre permet d'établir des relations entre les chercheurs, de faire l'exposé des recherches, d'aider les spécialistes dans leur ignorance réciproque, d'étendre le champ des applications (exemple : les tests) et de sauvegarder le titre de psychologue dont l'abus est manifeste de nos jours.

Un échange de vues se produit sur les modalités d'institution d'une Société de Psychologie sur le modèle des organismes étrangers de même nature. Une commission est constituée aux fins d'élaborer les statuts du nouveau groupement, commission composée des personnalités suivantes : MM. Michotte, Nyssen, Fransen, Nuttin et Paulus, secrétaire. Il est décidé que les statuts provisoires rédigés par cette commission seront adressés à tous les membres présents avant leur discussion publique.

La séance est levée à 12 h.

Séance du **Vergadering van**

15-II-1947

Présents : MM. Buyse, Coetsier, De Coster, Dellaert, Drabs,

De Greeff, Jonckheere, Michotte, de Montpellier, Nuttin, Paulus, Rijlant, Verheyen.

M. Michotte, président, ouvre la séance à 10 h. Il expose l'activité de la commission restreinte, chargée de l'élaboration des statuts provisoires de la société belge de psychologie. Les statuts ayant été adressés à tous les membres présents dans les deux langues nationales, lecture est donnée de ses divers articles avant leur rédaction définitive.

Divers articles sont modifiés après examen critique.

Le Bureau de la société est constitué ensuite. L'assemblée désigne à l'unanimité les membres suivants :

M. Michotte, Président, Professeur à l'Université de Louvain.

M. Aug. Ley, Vice-Président, Professeur honoraire à l'Université de Bruxelles;

M. De Coster, Secrétaire, chargé de cours à l'Université de Bruxelles;

M. Nuttin, Trésorier, Professeur à l'Université de Louvain;

M. Fransen : Professeur à l'Université de Gand;

M. Nihard, Professeur à l'Université de Liège.

Des échanges de vue ont lieu sur diverses questions, telles que : lieu des réunions, publication des travaux, impression des statuts, perceptiondes cotisations, abonnement aux revues étrangères, etc. Il est décidé que la première séance plénière sera consacrée à un exposé, par M. Michotte, de ses récents travaux, et qu'elle aura lieu au Laboratoire de M. Michotte à Louvain.

Séance du — Vergadering van

18-X-1947

La séance est ouverte à 14.30 h.

Monsieur Michotte van den Berck procède, en présence de l'Assemblée debout, à l'éloge funèbre de deux membres fondateurs décédés, Monsieur le professeur René Nihard (Université de Liège) et Monsieur le chargé de cours le Docteur Justinien Heernu (Université de Bruxelles).

Communication de M. Michotte van den Berck :

La perception de la causalité

Après avoir rappelé les théories classiques sur le problème

de la causalité, l'orateur montre que les progrès en psychologie n'ont lieu que lorsque les chercheurs découvrent la technique adéquate à les promouvoir. L'exploitation d'une technique nouvelle a permis à l'auteur de considérer le problème de la causalité non sous les aspects de la récognition et de l'association des idées, mais sous l'angle de la perception.

Il serait trop long, dans ce compte-rendu, de résumer la doctrine du conférencier, celle-ci ayant paru — par ailleurs — dans son volume relatif à la question. Des expériences nombreuses illustraient cette communication.

On servit ensuite le thé.

Après une interruption de 30 minutes, la séance reprit, consacrée aux activités suivantes :

1. Discussion de l'exposé. Des éclaircissements furent demandés et des critiques formulées. A ce débat prirent part : M. Barzin, invité, MM. De Coster, Dellaert, Fransen, Nuttin et Nyssen.

2. Recrutement de nouveaux membres. Le secrétaire est invité à adresser les statuts de la société à un certain nombre de personnes.

3. Projet de Michotte-Jeanjot. Un comité provisoire de psychologie est formé, comprenant les personnes suivantes : MM. Buyse, Drabs, Fauville, Fransen, Jonckheere, Nuttin et Nyssen. A l'unanimité la présidence en est confié à M. Michotte van den Berck.

4. Publications des membres. Diverses suggestions sont faites. Aucune conclusion positive ne sort de ce débat.

5. Fixation de la prochaine assemblée générale au 13 décembre.

Monsieur le Président lève la séance à 19 h. 30.

Séance du — Vergadering van

13-XII-1947

Monsieur le Président ouvre la séance à 14 h.30.

Il donne à l'Assemblée connaissance des décisions prises par le Bureau et lui demande de les approuver.
