# Supplementary material for: The Belgian Association for Psychological Sciences: 75th Anniversary
Source: Psychol Belg. 2022 Apr 28;62(1):184–92. doi: 10.5334/pb.1140 (PMC9053530; doi:10.5334/pb.1140)
Supplement: Documents related to the BAPS foundation Part 2. [file pb-62-1-1140-s2.pdf]

B. Het probleem van de psychologische onderbouw der vernieuwing. *Persoon en Gemeenschap*, 1947, nº 3/4.
Het probleem van de schooldifferentiatie in verband met de psychische mogelijkheden der kinderen. *Ibid.*, 1947, nº 3/4.
Meisjesopvoeding. *Ibid.*, 1947, nº 3/4.
(In samenwerking met Dr. paed. DE COSTER), Het bewegings-, arbeids-, en uitdrukkingsbeginsel in de nieuwere opvoeding. Algemene schets van het probleem. Publicatie in afzonderlijke brochure, 1951; en ook in *Persoon en Gemeenschap*.
De « Werkplaats-Kindergemeenschap » van Kees Boeke te Bilthoven. Publicatie in afzonderlijke brochure, alsook in *Vernieuwing*, 1951, September, nº 86.
Het buitengewoon onderwijs gezien van uit het standpunt der vernieuwing. *Vernieuwing*, Febr., 1953, nº 101.

WILLEMS, Emiel Frans : *secretaris van de Provinciale Centrale voor Beroepsoriëntering van Limburg.*

1. Tramstraat 6, Hasselt. — Tel. 230.02.
2. Weerstandlaan 8, Hasselt. — P.C.R. 4206.27.
3. º Vorselaar, 18-II-23. — Belg. Nat.
4. Lic. Paedagogische Wetenschappen (Univ. Leuven, 1948).
   Lic. B.O. & P.S. (Univ. Leuven, 1948).
   Lic. Criminologische Wetenschappen (Univ. Leuven, 1948) (verhandeling nog te presenteren).
5. De Geschiedenis van de Jeugdbeweging (Dir. : Prof. D'Espallier, 1948).
6. Lid van de Internationale Vereniging voor Psychotechniek. — Lid van de Katholieke Vereniging voor Geesteshygiëne. — Aangesloten lid van de Belgische Vereniging voor Psychologie.
7. Secretaris van de Prov. Centrale voor Beroepsoriëntering van Limburg (1949-).

Bibliographie :

B. Sociometrie. *C.B.O.-Periodiek*, 1949, nº 3-4, 6/1-6/6.
Het IXᵉ Internationaal Congres voor Psycho-techniek te Bern van 12 tot 17 September. *Ibid.*, November 1949, 10/1-10/2.
Welk is de invloed van de 6ᵉ proef van de Coetsier-schaal op de intelligentieindex ? *Ibid.*, 1950, nº 6, 20/1-20/2.
Bij « Personality » door Gardner Murphy. *Ibid.*, 1950, nº 2, 7/1-7/5.
Jeugdbeweging, geschiedenis. *Kath. Encyclopaedie voor Onderwijs en Opvoeding*, Vol. 2. 1952, 376-379.
Jeugdbeweging, België. *Ibid.*, 381-385.
Jeugdbeweging, De K.A.J. *Ibid.*, 385-386.
Jeugdbeweging, F.E., Jeugdbeweging, K.S.A. - Jong-Vlaanderen. *Ibid.*, 389.
Jeugdzorg. *Ibid.*, 421-422.

TROISIÈME PARTIE

DERDE DEEL

DOCUMENTS ET CHRONIQUE
DOCUMENTEN EN KRONIEK

I. — LISTE DES MEMBRES DE LA SOCIETE
LEDENLIJST VAN DE VERENIGING

(31-XII-1953)

A. *Président d'honneur — Ere-Voorzitter* :

A. Michotte van den Berck.

B. *Bureau — Bestuur* (1952-1955)

Président - Voorzitter : R. Nyssen.
Vice-Président - Ondervoorzitter : J. Paulus.
Secrétaire - Secretaris : J. Nuttin.
Trésorière - Schatbewaarster : J. Declerck.
C.C.P. - P.C.R. : Nº 51.40.26 (Mlle J. Declerck, Rixensart).
Délégués - Afgevaardigden : L. Coetsier, L. Delys.

C. *Membres titulaires - Werkende Leden* :

1. Membres fondateurs - Stichtende Leden :

MM. N. Braunshausen
R. Buyse
L. Coetsier
S. De Coster
E. De Greeff
R. Dellaert
G. de Montpellier d'Annevoie
V. D'Espallier
J. Drabs
A. Fauville
F. Fransen

MM. T. Jonckheere
A. Kriekemans
A. Ley
A. Michotte van den Berk
J. Nuttin
R. Nyssen
J. Paulus
R. Piret
P. Rijlant
J. von Schmid

2. Autres membres titulaires — Andere werkende Leden :

M. J. De Busscher
Mlle J. De Clerck
MM. A. Delvaux
L. Delys
F. Hotyat
Mme J. Jadot-Decroly
M. L. Knops
Mme M. L. Ley-Wauthier

MM. L. Litwinski
C. Mertens de Wilmars
A. Ombredane
P. Osterrieth
R. Pasquasy
J. Titeca
W. Tordeur
A. Van Wayenberghe

D. *Membres adhérents — Aangesloten Leden* :

MM. J. Bourdon
R. De Craecker
R. Derivière
J. Dierkens
J. Flament
G. Goosens
H. Haroux
Mlle M. Leblanc
M. J. Segers

MM. W. Smet
F. Stienlet
J. Stinissen
A. Thienpont
G. Thinès
W. Van Hove
A. Van Petegem
Mej. M. Wens
M. E. Willems

II. — STATUTS DE LA SOCIETE
STATUTEN VAN DE VERENIGING

Article premier. — Il est constitué une *Société Belge de Psychologie.* Son siège est à Bruxelles.

Son but est de grouper tous ceux qui, en Belgique, font de la psychologie, entendue comme une discipline strictement positive et scientifique, l'objet de leurs recherches théoriques ou de leur activité pratique et professionnelle. Elle vise à susciter entre eux des échanges de vue, à coordonner leurs travaux, enfin à veiller à la sauvegarde de leurs intérêts professionnels ainsi qu'à celle du statut de psychologue.

Artikel 1. — Er bestaat een *Belgische Vereniging voor Psychologie,* waarvan de zetel gevestigd is te Brussel.

De Vereniging heeft tot doel al diegenen in België te groeperen voor wie de psychologie, als positieve wetenschap, het voorwerp uitmaakt van hun theoretische onderzoekingen of van hun practische beroepsactiviteit. Zij beoogt onder haar leden uitwisseling van gedachte en samenordening van werk mogelijk te maken, alsook te waken op de beroepsbelangen en het statuut van de psycholoog.

Art. 2. — La société belge de Psychologie comprend :

1° des membres titulaires, dont le nombre est limité à 50;

2° des membres adhérents, dont le nombre est illimité.

Toute candidature au titre de membre adhérent doit être présentée par deux membres titulaires et transmise au Bureau. A la suite de la demande, le Bureau constitue une Commission d'au moins trois membres, dont le Président, qui fait rapport, dans les trois mois, sur la valeur morale du candidat et sur ses titres scientifiques.

Elle exigera en principe :

1° que le candidat possède un

Art. 2. — De Belgische Vereniging voor Psychologie telt :

1° werkende leden, waarvan het aantal tot 50 beperkt is;

2° aangesloten leden waarvan het aantal niet beperkt is.

Elke candidatuur tot aangesloten lid van de Vereniging moet voorgedragen worden door twee werkende leden en overgemaakt worden aan het Bestuur. Als gevolg op de aanvraag wordt een Commissie aangesteld bestaande uit drie leden (waaronder de Voorzitter), die binnen de drie maand verslag uitbrengt over de morele waarde en de wetenschappelijke titels van de candidaat.

In principe wordt van de candidaat geëist :

diplôme d'études universitaires complètes;

2° qu'il exerce, dans le domaine de la psychologie scientifique, une pratique jugée suffisante par la Commission.

La Commission de candidature présente son rapport devant le Bureau. Les conditions d'admissibilité étant remplies, la candidature est portée devant l'Assemblée et il est procédé au vote par scrutin secret. Pour être admis, le candidat doit réunir les deux tiers des voix des membres (titulaires et adhérents) présents à la séance.

Les membres adhérents ont le droit de participer aux activités de la Société (conférences, discussions, communications). Ils paient la même cotisation et ont droit aux mêmes avantages matériels (publications éventuelles, etc.) que les membres titulaires. Ils sont représentés au sein du Bureau par un de leurs membres spécialement élu par leur groupe. Ils ont droit de vote, sauf sur les trois points suivants concernant lesquels ils ont voix consultative :

1° Changement des Statuts;

2° Nomination des membres titulaires;

3° Election du Bureau, à l'exception du délégué des membres adhérents.

Pour être nommé membre titulaire il faut :

1° que le candidat exerce une

1° Dat hij een diploma van volledige universitaire studiën bezit;

2° Dat hij gedurende een tijd die door de Commissie voldoende wordt geacht op psychologisch gebied werkzaam is in een wetenschappelijk verantwoorde practijk.

De Commissie brengt verslag uit voor het Bestuur. Wanneer de candidaat aan de voorwaarden ter aanvaarding voldoet, wordt zijn candidatuur aan de Vergadering voorgelegd en gaat men over tot de geheime stemming. De candidaat wordt aangenomen wanneer hij door de twee derden van alle aanwezige leden (werkende en aangeslotene) wordt gekozen.

De aangesloten leden hebben recht tot deelname aan de activiteiten van de Vereniging (voordrachten, mededelingen, discussies, enz.). Zij betalen dezelfde jaarlijkse bijdrage en genieten dezelfde materiële voordelen (eventuele publicaties, enz.) als de werkende leden. Zij zijn vertegenwoordigd in het Bureau door één van hun leden die speciaal door hun groep wordt gekozen. Zij hebben stemrecht, uitgenomen in de volgende drie aangelegenheden, waarin zij alleen adviserend kunnen tussenkomen.

1° Wijziging van de Statuten.

2° Benoeming van werkende leden.

3° Verkiezing van de leden van het Bureau, met uitzondering van de vertegenwoordiger van de aangesloten leden.

activité importante dans le domaine de la psychologie et qu'il ait à son actif des travaux d'une valeur scientifique reconnue;

2° qu'il soit présenté par le Bureau;

3° qu'il soit élu à la majorité des deux tiers des membres titulaires présents.

Les nouveaux membres seront, normalement, admis d'abord comme membres adhérents; la promotion au titre de membre titulaire se fera, éventuellement, après plusieurs années d'activité scientifique.

La Société peut aussi désigner des membres d'honneur sur une proposition unanime du Bureau, l'élection étant faite à la majorité des deux tiers des membres titulaires et adhérents présents à la séance. Le nombre des membres d'honneur ne dépassera pas 10.

Art. 3. — Les membres versent une cotisation annuelle dont le montant est fixé chaque année par le Bureau moyennant l'accord de l'Assemblée. Cette cotisation donne droit aux publications ordinaires de la Société.

Art. 4. — Tout membre qui néglige d'acquitter sa cotisation, se voit supprimer le service des publi-

Om aangenomen te worden als werkend lid is vereist :

1° Dat de candidaat een belangrijke activiteit uitoefent op psychologisch gebied en dat hij werk heeft geleverd waarvan het wetenschappelijk belang algemeen erkend is.

2° Dat zijn candidatuur door het Bestuur wordt voorgedragen.

3° Dat hij gekozen wordt door twee derden van de aanwezige werkende leden.

Nieuwe leden zullen in algemene regel eerst als aangesloten leden worden aangenomen. De eventuele promotie tot werkend lid zal slechts na meerdere jaren wetenschappelijke activiteit kunnen gebeuren.

De Vereniging kan ook ereleden benoemen. De benoeming gebeurt op het eensgezinde voorstel van het Bestuur en door een verkiezing waarbij de candidaat de twee derden der stemmen van alle aanwezige leden (werkende en aangesloten leden) bekomt. Het aantal ereleden zal de tien niet te boven gaan.

Art. 3. — De leden storten een jaarlijkse bijdrage, waarvan het bedrag ieder jaar door het Bestuur met goedkeuring van de Vergadering wordt vastgesteld. Deze bijdrage geeft recht op de gewone publicaties van de Vereniging.

Art. 4. — De publicaties worden niet verder toegezonden aan het lid dat zijn bijdrage niet betaalt. De

cations. Le Trésorier communiquera au Secrétaire les noms des membres qui ont refusé d'acquitter le montant de leur cotisation, même après présentation d'une quittance postale. Cette quittance est majorée de 10 % pour frais d'écriture et d'encaissement. Le membre qui refusera d'acquitter sa cotisation sera averti par lettre recommandée avec avis de réception que sa négligence entraînera sa radiation de la liste des membres. S'il n'est pas donné suite favorable à cette lettre, la radiation sera prononcée *ipso facto*.

Art. 5. — Tout membre qui aura porté atteinte à l'honneur de la Société ou de ses membres sera l'objet d'une sanction (blâme, exclusion temporaire ou définitive). La sanction ne sera prononcée qu'après une séance à huis clos, où le membre incriminé, dûment convoqué par lettre recommandée, sera entendu dans ses moyens de défense. En cas de non-comparution, il sera passé outre et la Société pourra se prononcer séance tenante.

Le vote a lieu au scrutin secret. L'exécution ne peut être prononcée que moyennant les deux tiers des voix des membres présents. Le procès verbal de cette séance est conservé dans les archives de la Société. Il n'est pas publié.

Art. 6. — La Société est dirigée

Schatbewaarder zal aan de Secretaris de namen overmaken van de leden die, zelfs na aanbieding van een postkwitantie, hun bijdrage niet betalen. Het bedrag van de kwitantie wordt met 10 % verhoogd tot dekking van de onkosten. Het lid dat zijn bijdrage weigert te betalen zal per aangetekende brief, met kennisgeving van ontvangst, verwittigd worden, dat zijn nalatigheid de royering als lid voor gevolg zal hebben. Indien aan dit schrijven geen gunstig gevolg wordt gegeven, wordt de royering *ipso facto* uitgesproken.

Art. 5. — Elk lid dat een aanslag pleegt op de eer van de Vereniging of van haar leden, zal het voorwerp zijn van een sanctie (blaam, tijdelijke of definitieve uitsluiting). De uitsluiting zal eerst worden uitgesproken nadat het lid, opgeroepen per aangetekende brief, de gelegenheid kreeg om in een vergadering met gesloten deuren, zijn verdediging op zich te nemen.

Wanneer het lid niet verschijnt, kan de Vereniging in de vergadering zelf uitspraak doen. De stemming geschiedt geheim. De uitsluiting kan alleen gebeuren op grond van twee derden der stemmen van de aanwezige leden. Het proces verbaal van deze zitting wordt in het archief der Vereniging bewaard; het wordt niet gepubliceerd.

Art. 6. — De leiding van de

par un Bureau composé d'un Président, d'un Vice-Président, d'un Secrétaire, d'un Trésorier, et de deux Délégués. Un des deux délégués est élu parmi les membres adhérents.

Le Bureau est élu en Assemblée générale à la majorité absolue des membres titulaires votants. Le délégué des membres adhérents est élu à la majorité absolue des membres adhérents votants. Les votes par correspondance sont admis.

Le mandat est de quatre ans. Les membres du Bureau ne sont pas immédiatement rééligibles au poste qu'ils occupent, à l'exception du Secrétaire et du Trésorier.

En cas de décès ou de démission de l'un de ses membres, le Bureau peut se compléter par cooptation, pour achever le mandat en cours. Son choix doit être ratifié par la prochaine Assemblée générale.

Art. 7. — La Société tient une réunion au moins tous les deux mois au cours de l'année académique, soit au minimum quatre réunions par an. L'emploi des deux langues nationales est libre.

Au cours de ces réunions, on entendra notamment des conférences des membres ou de certaines personnes invitées sur des questions

Vereniging is toevertrouwd aan een Bestuur, bestaande uit een Voorzitter, een Onder-Voorzitter, een Secretaris, een Schatbewaarder en twee Afgevaardigden waarvan één gekozen wordt onder de aangesloten leden.

Het Bestuur wordt gekozen in Algemene Vergadering met de absolute meerderheid van de stemmende werkende leden. De afgevaardigde van de aangesloten leden word gekozen met de absolute meerderheid van de stemmende aangesloten leden. Stemming per brief is toegelaten.

Het mandaat loopt over vier jaar. De Bestuursleden zijn niet onmiddellijk herkiesbaar in de functie die zij bekleden, met uitzondering van de Secretaris en de Schatbewaarder.

Bij overlijden of ontslag van één zijner leden, kan het Bestuur door cooptatie zijn ledenaantal aanvullen, ten einde de lopende opdracht te voleinden. Zijn keuze moet op de volgende Algemene Vergadering worden bekrachtigd.

Art. 7. — De Vereniging vergadert minstens om de twee maand gedurende het academiejaar, dit is ten minste viermaal per jaar. Het gebruik der twee nationale talen is vrij.

Op deze vergaderingen zullen onder andere voordrachten worden gehouden door de leden, of door genodigden, over een psychologisch

d'ordre psychologique. La durée de ces conférences ne peut pas, en général, dépasser trente minutes, de façon qu'une discussion utile puisse suivre. Les membres qui désirent faire une conférence aux séances, en adressent un bref résumé au Bureau, qui avise le Sociétaire de l'acceptation de sa conférence, ainsi que de la date qui peut lui être assignée.

De plus, les membres sont autorisés à présenter, aux séances, de courtes notes sur des questions qu'ils croient de nature à intéresser la Société, à la condition de s'être mis d'accord au préalable avec le Bureau à ce sujet. La durée de ces communications ne pourra dépasser dix minutes.

Art. 8. — Lors de la première réunion de l'année académique, le Secrétaire fait rapport sur l'activité de la Société au cours de l'année écoulée. Le Trésorier soumet à l'Assemblée les comptes de recettes et dépenses et le budget.

Art. 9. — Toute modification aux statuts doit être soumise à une Assemblée générale spécialement convoquée à cet effet. Elle ne peut être acceptée que moyennant le consentement des deux tiers des

onderwerp. De duur van deze voordrachten zal in de regel de dertig minuten niet overschrijden, zodat tijd voor een nuttige gedachtenwisseling beschikbaar blijft. De leden die op de vergadering een voordracht wensen te houden, sturen een korte samenvatting er van naar het Bestuur. Het Bestuur geeft aan het lid bericht over de aanvaarding van zijn voordracht en over de datum waarop deze zal kunnen plaats hebben.

Daarenboven is het aan de leden toegelaten op de vergaderingen korte mededelingen te doen over onderwerpen waarvan zij menen dat ze de Vereniging kunnen interesseren. Vooraf moet het lid hierover een afspraak maken met het Bestuur. Deze korte mededelingen zullen niet meer dan tien minuten duren.

Art. 8. — Op de eerste vergadering van het academiejaar, brengt de Secretaris verslag uit over de werkzaamheden van de Vereniging gedurende het afgelopen jaar. De Schatbewaarder legt aan de Vergadering het uittreksel voor van uitgaven en inkomsten en van het budget.

Art. 9. — Elke wijziging aan de statuten moet aan de Algemene Vergadering onderworpen worden in een bijeenkomst tot dat doel. De wijziging wordt aanvaard wanneer zij door de twee derden der stem-

membres titulaires votants. Les votes par correspondance sont admis. Pour être prise en considération, la dite modification doit être proposée par dix membres au moins ou émaner du Bureau.

Art. 10. — La dissolution de la Société ne peut être prononcée que par une Assemblée générale réunissant les conditions et suivant la procédure prescrites à l'art. 9. Les fonds qui resteraient à l'actif de la Société seraient affectés à une œuvre d'intérêt psychologique.

mende werkende leden wordt goedgekeurd. De stemming per brief is toegelaten.

Om in aanmerking te komen moet de voorgestelde wijziging uitgaan van minstens tien leden ofwel van het Bestuur.

Art. 10. — De ontbinding kan alleen worden uitgesproken door de Algemene Vergadering in een bijeenkomst en volgens een procedure als aangeduid in art. 9. De fondsen die de Vereniging nog op haar actief zou hebben, zouden op een of ander psychologisch werk worden overgedragen.

III. — DOCUMENTS POUR SERVIR A L'HISTOIRE DE LA PSYCHOLOGIE EN BELGIQUE
DOKUMENTEN VOOR DE GESCHIEDENIS VAN DE PSYCHOLOGIE IN BELGIE

Le Fondateur de la Société belge de Psychologie, M. le Professeur A. Michotte van den Berck, a été invité par le Comité de rédaction de l'*History of Psychology in Autobiography* (Clark University Press) à publier une autobiographie scientifique dans le quatrième volume de cet ouvrage qui vient de paraître en 1953.

Le Bureau de la Société a obtenu la permission des éditeurs de publier ici pour la première fois le texte français original de cette autobiographie qui est de nature à intéresser

De Stichter van de Belgische Vereniging voor Psychologie, Professor A. Michotte van den Berck, werd door de redactie van het verzamelwerk *A History of Psychology in Autobiography* uitgenodigd een wetenschappelijke autobiographie te publiceren in het vierde deel van dit werk.

Het Bureau van onze Vereniging heeft van de uitgevers de toelating verkregen om de oorspronkelijke franse tekst van deze autobiographie voor het eerst te publiceren. Dit werk is van dien aard dat het ieder-

tous ceux qui désirent connaître la psychologie scientifique en Belgique.

C'est à ce titre qu'elle est publiée ici. Le Bureau espère pouvoir publier ultérieurement des documents similaires pour d'autres centres de psychologie.

een, die met de wetenschappelijke psychologie in Belgie heeft te maken, zal interesseren. Om die reden wordt het hier gepubliceerd. Het Bestuur hoopt dat het zal mogelijk zijn in de toekomst gelijkaardige documenten te publiceren voor andere psychologische centra.

AUTOBIOGRAPHIE DE M. LE PROFESSEUR A. MICHOTTE van den BERCK

Lorsqu'on voulut bien me proposer de publier mon autobiographie, mon premier mouvement fut de décliner cet honneur. Ma vie, tant professorale et scientifique que familiale s'est en effet déroulée d'une façon si simple, si régulière, si logique pourrait-on dire, qu'elle me paraissait présenter bien peu d'intérêt pour des personnes n'appartenant pas à mon entourage immédiat.

Puis, à la réflexion, j'ai pensé qu'il était peut-être souhaitable après tout de voir figurer dans cette collection biographique les échantillons les plus variés des « styles de vie » des psychologues de notre époque, et j'ai cédé à l'aimable insistance de quelques collègues.

« Styles de vie » peut signifier bien des choses, mais il va sans dire que c'est uniquement de la vie scientifique qu'il est question dans ma pensée car, étant donné qu'il s'agit en l'occurrence d'une contribution à l'Histoire de la Psychologie (et même à sa « petite histoire »), c'est surtout l'apport personnel à nos connaissances en cette matière qui importe me semble-t-il, ainsi que l'évolution individuelle des idées, et la recherche des raisons qui l'ont provoquée.

Tel est l'esprit dans lequel ont été conçues ces pages (1).

Je suis né en 1881 à Bruxelles, second et dernier enfant d'une famille d'intellectuels fortunés, qui s'intéressaient par tradition aux beaux-arts

(1) Je me permets de signaler qu'une courte biographie ainsi que des indications bibliographiques complètes et une liste de tous les travaux entrepris à mon laboratoire ont été publiées dans les *Miscellanea Psychologica Albert Michotte*, pp. I-XL éditées en 1947, à l'occasion de mon jubilé professoral, par les Editions de l'Institut Supérieur de Philosophie, à Louvain.

ainsi qu'aux sciences, aussi ma jeunesse s'est-elle écoulée dans un milieu de haute culture (2). Tous mes ascendants connus étaient catholiques et sincèrement religieux, comme je l'ai été moi-même durant toute ma vie.

Je suis entré en 1897 comme étudiant à l'Université de Louvain où j'ai étudié la philosophie et les sciences jusqu'en 1905. J'eus la chance d'y rencontrer deux grands maîtres : l'homme incomparable qui devait devenir l'illustre Cardinal Mercier et qui était alors professeur et Président de l'Institut Supérieur de Philosophie. Celui-ci voulut bien dès le début me témoigner une sollicitude et une affection toutes paternelles et son influence fut déterminante dans ma formation intellectuelle et « humaine », et j'en ressens aujourd'hui encore les effets bienfaisants.

Le second était le grand neurologue Arthur van Gehuchten qui m'initia au travail scientifique, et sous la direction duquel je fis mes premières recherches (3).

Mercier s'intéressait vivement à la Psychologie, aussi lorsqu'il fonda l'Institut de Philosophie, voulut-il y introduire l'enseignement de la psychophysiologie. Il s'était assuré à cet effet la collaboration du professeur Armand Thiéry qui avait travaillé chez Wundt à Leipzig et qui organisa dès 1894 un petit laboratoire de Psychologie au dit Institut. C'est là que j'appris les premiers rudiments de notre science. Toutefois, Thiéry très préoccupé de questions philosophiques et théoriques n'était pas un homme de laboratoire, aussi lorsque ma formation tant philosophique que scientifique fut suffisamment poussée, Mercier, m'encouragea-t-il fortement à poursuivre le dessein que j'avais formé de me spécialiser en Psychologie. J'entrepris alors sous la direction de Thiéry des recherches sur la répartition de la sensibilité tactile (4).

Puis je me rendis à Leipzig où je passai deux semestres en 1905 et 1906 et c'est là en somme que je commençai réellement à me familiariser avec la Psychologie car le laboratoire de Louvain était encore embryonnaire et sa bibliothèque l'était davantage... Je me suis imprégné à ce moment des « Grundzüge der Physiologischen Psychologie » et me suis initié à la technique expérimentale. J'avais une très grande admiration pour

(2) Mon frère Paul fut également professeur à l'Université de Louvain où il enseigna la Géographie et fonda un Institut de Géographie qui porte son nom.

(3) Michotte A., Contribution à l'étude de l'histologie fine de la cellule nerveuse. Bulletin de l'Académie Royale de Médecine de Belgique. 1904, pp. 515-556.

(4) Michotte A., *Les Signes Régionaux*. Institut Supérieur de Philosophie. Louvain, 1905; 195 pp.
